# A mutual information measure of phase-amplitude coupling using gamma generalized linear models

**DOI:** 10.3389/fncom.2024.1392655

**Published:** 2024-05-22

**Authors:** Andrew S. Perley, Todd P. Coleman

**Affiliations:** Department of Bioengineering, Stanford University, Stanford, CA, United States

**Keywords:** electrophysiology, cross-frequency coupling, generalized linear model, goodness-of-fit, gut-brain coupling

## Abstract

**Introduction:**

Cross frequency coupling (CFC) between electrophysiological signals in the brain is a long-studied phenomenon and its abnormalities have been observed in conditions such as Parkinson's disease and epilepsy. More recently, CFC has been observed in stomach-brain electrophysiologic studies and thus becomes an enticing possible target for diseases involving aberrations of the gut-brain axis. However, current methods of detecting coupling, specifically phase-amplitude coupling (PAC), do not attempt to capture the phase and amplitude statistical relationships.

**Methods:**

In this paper, we first demonstrate a method of modeling these joint statistics with a flexible parametric approach, where we model the conditional distribution of amplitude given phase using a gamma distributed generalized linear model (GLM) with a Fourier basis of regressors. We perform model selection with minimum description length (MDL) principle, demonstrate a method for assessing goodness-of-fit (GOF), and showcase the efficacy of this approach in multiple electroencephalography (EEG) datasets. Secondly, we showcase how we can utilize the mutual information, which operates on the joint distribution, as a canonical measure of coupling, as it is non-zero and non-negative if and only if the phase and amplitude are not statistically independent. In addition, we build off of previous work by Martinez-Cancino et al., and Voytek et al., and show that the information density, evaluated using our method along the given sample path, is a promising measure of time-resolved PAC.

**Results:**

Using synthetically generated gut-brain coupled signals, we demonstrate that our method outperforms the existing gold-standard methods for detectable low-levels of phase-amplitude coupling through receiver operating characteristic (ROC) curve analysis. To validate our method, we test on invasive EEG recordings by generating comodulograms, and compare our method to the gold standard PAC measure, Modulation Index, demonstrating comparable performance in exploratory analysis. Furthermore, to showcase its use in joint gut-brain electrophysiology data, we generate topoplots of simultaneous high-density EEG and electrgastrography recordings and reproduce seminal work by Richter et al. that demonstrated the existence of gut-brain PAC. Using simulated data, we validate our method for different types of time-varying coupling and then demonstrate its performance to track time-varying PAC in sleep spindle EEG and mismatch negativity (MMN) datasets.

**Conclusions:**

Our new measure of PAC using Gamma GLMs and mutual information demonstrates a promising new way to compute PAC values using the full joint distribution on amplitude and phase. Our measure outperforms the most common existing measures of PAC, and show promising results in identifying time varying PAC in electrophysiological datasets. In addition, we provide for using our method with multiple comparisons and show that our measure potentially has more statistical power in electrophysiologic recordings using simultaneous gut-brain datasets.

## 1 Introduction

Neurons in the body operate by communicating with other neurons, often in circuits. It's been hypothesized that these neuronal circuits can give rise to coupling of local field potentials (LFPs) that are observed via various methods of electroencephalography. This coupling phenomenon can manifest itself in the form of cross-frequency coupling (CFC), in which different frequency bands in the LFPs of certain brain regions modulate LFPs in the same or different brain regions (Canolty and Knight, 2010). It has further been hypothesized that CFC underlies modulation of neural excitability and is a driver of long-range communication in the brain (Canolty and Knight, 2010). One commonly observed form of CFC is phase-amplitude coupling (PAC), and is characterized by the modulation of the amplitude of a higher frequency signal by the phase of a lower frequency signal. This is somewhat akin to the common communication scheme, amplitude modulation (AM), that is used in radio. Early reports of this phenomena showed that the amplitude of the gamma band activity in the hippocampus of a rat was tied to different phases of the theta band activity (Bragin et al., 1995). PAC has also been seen as a powerful biomarker to investigate stress, depression, Parkinson's disease, and epilepsy amongst other conditions (De Hemptinne et al., 2013; Edakawa et al., 2016; Wang et al., 2021).

Recent work involving brain-viscera electrophysiologic dynamics has demonstrated PAC between the gastric slow wave (0.05 Hz) and the alpha band (8–12 Hz) of certain cortical regions, including the right anterior insula and occipital-parieto regions (Richter et al., 2017). This finding has potential to allow for new characterizations of medical conditions involving changes in the gut-brain axis, including depression, autism spectrum disorders, and obesity (Fülling et al., 2019). However, despite recent advancements and electrophysiological coupling being an enticing early biomarker for studying health and physiology, the current most common techniques for assessment of PAC have not changed significantly (Hülsemann et al., 2019). Additionally, work in development in PAC measures has traditionally been done in the context of the brain and there are no studies that directly assess the validity of such measures in very low frequency signals like the gastric slow wave. These two facts lead us to survey existing methods and develop our own metric of PAC.

The most commonly used method of PAC is the Modulation Index (MI) (Tort et al., 2010). This measure has been shown to be efficient, fast, and reliable in calculating PAC, but trades off some statistical rigor for efficiency. Other work in development of PAC measures includes normalized direct PAC (ndPAC), which aims to keep such simplicity, but also include statistical thresholds for assessing significant coupling (Özkurt, 2012). More recent developments in PAC also include methods to assess time-resolved PAC phenomena that utilize things such as block experiment design, time-frequency methods, or state-space models (Voytek et al., 2013; Munia and Aviyente, 2019; Soulat et al., 2022). In addition, information theoretic quantities have also found their way into PAC estimation. In particular, non-parametric estimation of mutual information has become of interest and shows promise in assessment of transient PAC, but tends to generate biased estimates (Martinez-Cancino et al., 2019).

In this work, we develop a new mutual information measure of PAC and validate it using simulated gut-brain signals and intracranial EEG recordings. We use a convex parametric method to fitting the joint probability distribution on the phase and amplitude of two electrophysiologic signals and then subsequently calculate the mutual information as our measure of PAC. Specifically, we develop a gamma generalized linear model (GLM) to model the conditional distribution of amplitude given phase and use a Fourier basis of regressors as a natural basis to fit our model. We use the minimum description length (MDL) principle to choose the optimal number of bases to fit our model (Barron et al., 1998). We utilize the Kolmogorov–Smirnov test as a method for assessing the goodness-of-fit (GOF) of the model and demonstrate exemplary GOF on human EEG datasets (Massey Jr., 1951).

We calculate the mutual information using Bayes' rule and numerical integration over the circle, which allows for reliable numerical estimates of the mutual information. Our use of a parametric method allows for exact estimates of mutual information given that we have adequate model fit (Ince et al., 2022). We then validate our PAC statistic by generating synthetic phase-amplitude coupled signals to mimic gut-brain coupling and perform an ROC curve analysis against the prevailing common methods of assessing PAC. Furthermore, we test our PAC statistic in mouse intracranial electroencephalography (iEEG) data to replicate past findings of PAC using comodulograms. To demonstrate our technique's potential in gut-brain electrophysiology, we also generate topoplots of the PAC between simultaneous 128-channel scalp EEG and electrogastrography (EGG) recordings. To further understand time-varying dynamics of PAC, we extend our method to consider information density as a time-varying measure of PAC as in the seminal work by Martinez-Cancino et al. (2019), and use that to evaluate event-related PAC, for event related potential (ERP)-like phenomena as in Voytek et al. (2013). We evaluate both of these methods on synthetically generated coupled data with time-varying coupling. After validation in synthetic data, we then apply our methods to sleep spindle data and mismatch negativity (MMN) ERPs and demonstrate the potential for our methods to estimate time-varying PAC in human electrophysiology.

## 2 Methods

### 2.1 Preliminaries

#### 2.1.1 Definitions

Let *Y* ∈ [0, ∞) be a non-negative random variable that represents the amplitude of the fast neural signal and Θ ∈ [−π, π) be a circular random variable that represents the phase of the slow neural signal. We define the marginal probability densities on *Y* and Θ as *f*_*Y*_(*y*) and *f*_Θ_(θ). Furthermore, we assume that the density on Θ is uniform:


(1) $$\begin{array}{l} f_{\Theta }\left(\theta \right)=\frac{1}{2\pi },\;\theta \in \left[-\pi ,\pi \right]. \end{array}$$

This makes intuitive sense because Θ represents the phase of a narrowband oscillatory signal, which has approximately uniform phase over its support. We will use this assumption later in our calculation of our PAC metric. We also define *t* = 0, 1, …, *T* − 1, to be the time index of observed time series samples, and *Y*_*t*_ and Θ_*t*_ to be random variables with the same properties as *Y* and Θ, but observed at time *t*.

#### 2.1.2 Calculation of phase and amplitude

Given a one-dimensional signal *x*(*t*), we can calculate both its phase and amplitude using the Hilbert Transform. The Hilbert Transform of any signal is given by the convolution


$$\begin{array}{l} H\left\{x\left(t\right)\right\}=\frac{1}{\pi t}*x\left(t\right)\\ =\int_{-\infty }^{\infty }\frac{1}{\pi \left(t-\tau \right)}x\left(\tau \right)d\tau . \end{array}$$

This operation rotates the positive frequency components of the Fourier Transform of *x*(*t*) by –90 degrees and the negative frequency components by +90 degrees. This allows us to construct the analytic signal, a complex representation of *x*(*t*), as


$$\begin{array}{l} z\left(t\right)=x\left(t\right)+jH\left\{x\left(t\right)\right\}, \end{array}$$

which preserves the exact same information as *x*(*t*), but only using its positive frequency components. The power in this representation is that it allows us to write *z*(*t*) as a complex exponential


$$\begin{array}{l} z\left(t\right)=A\left(t\right)e^{j\phi \left(t\right)}, \end{array}$$

where we call *A*(*t*) the instantaneous amplitude of *x*(*t*), and ϕ(*t*) the instantaneous phase of *x*(*t*). In the case of a sinusoid of a single frequency, *A*(*t*)*sin*(ω*t* + ϕ), these two quantities represent the time-varying amplitude, *A*(*t*), and the argument of the sinusoid, ω*t* + ϕ, respectively. These two quantities are commonly used in computational neuroscience as estimates of the amplitude and phase of a neural oscillation, and are the quantities we will use in this paper.

#### 2.1.3 Mutual information as a measure of PAC

In order to assess the PAC between two electrophysiological signals, we consider the information theoretic quantity of mutual information (Gray, 2011). The mutual information between two random variables *X* and *Y*, with marginal distributions *P* and *Q* and joint distribution Γ, is most commonly given by


$$\begin{array}{l} I\left(X;Y\right)=D_{KL}\left(\Gamma ||P⊗Q\right), \end{array}$$

where *D_KL_*(·||·) is the relative entropy (a.k.a. the Kulback-Liebler Divergence) between two distributions. The distribution *P* ⊗ *Q* is the product of the marginal distributions and represents the joint distribution if *X* and *Y* are statistically independent. Note that the mutual information is always non-negative and exactly zero if and only if the two random variables are independent. This quantity is often interpreted as the amount of information one gains about *X* by observing *Y* or vice versa. The mutual information can account for nonlinearities in data, as opposed to the more commonly known correlation coefficient, which only captures linear relationships. These properties make the mutual information an exemplary candidate for assessment of PAC. In the following sections, we discuss how we use a parametric model to fit these distributions to data.

### 2.2 Gamma GLM

#### 2.2.1 Construction of the GLM

Generalized linear models (GLMs) are powerful tools often used for regression when the independent and/or dependent variables don't follow a normal distribution. Their construction is such that some function of the conditional expectation of the independent variable is modeled as a linear function of the dependent variable(s). This can be written as


(2) $$\begin{array}{l} g\left(\text{E}\left[Y|\Theta =\theta \right]\right)=R{\left(\theta \right)}^{⊤}w, \end{array}$$

where *g*(·) is called the link function, *R*(Θ)^⊤^ is a vector of regressors (function of dependent variables), and *w* is a vector of weights to be estimated.

**Figure 1:**
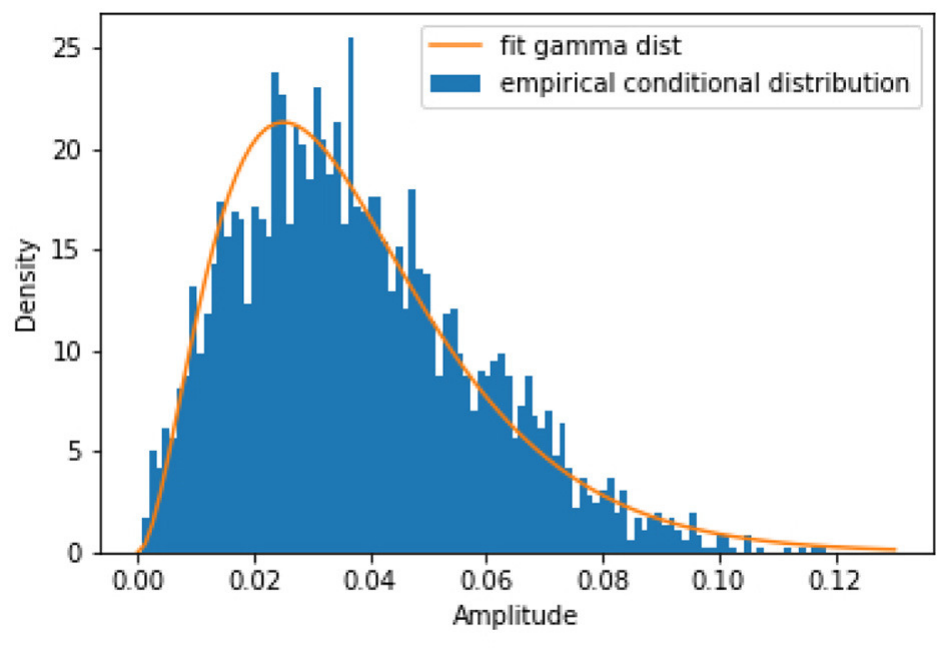
An exemplary plot of the empirical conditional distribution of the amplitude given phase from anesthetized mouse iEEG data in the hippocampal CA1 region. This shows that the gamma distribution is a good candidate for our model fitting procedure.

For GLMs, the conditional distribution must be of the exponential family (which includes the normal distribution) (McCullagh, 2019). Since the amplitude, *Y*, is non-negative, the normal distribution is unnatural. We turn to a flexible distribution of a non-negative random variable in the exponential family: the gamma distribution. Figure 1 shows an exemplary plot of the conditional distribution in iEEG data (from the hippocampal CA1 region in anesthetized mice) and its fit to the gamma distribution, demonstrating this as a sensible modeling choice (Scheffer-Teixeira et al., 2011). We generated the empirical conditional distribution by first generating a 2D histogram of amplitude and phase, with 100 phase and 100 amplitude bins. Then we selected, at random, a phase bin (in Figure 1 we selected bin 2) and took all the amplitudes associated to that phase bin and used that as the data for which we plotted a 1D histogram of amplitude given the specified phase bin.

The gamma distribution is a two-parameter distribution, with parameters α and β, given by the following form:


(3) $$\begin{array}{l} f_{Y}\left(y;\alpha ,\beta \right)=\frac{\beta^{\alpha }}{\Gamma \left(\alpha \right)}y^{\alpha -1}e^{-\beta y}, \end{array}$$

with expectation given by


(4) $$\begin{array}{l} E\left[Y\right]=\frac{\alpha }{\beta }. \end{array}$$

This implies that from Equations (2, 4), we can model our problem with


$$\begin{array}{l} g\left(\frac{\alpha }{\beta }\right)=R{\left(\theta \right)}^{⊤}w. \end{array}$$

Since *Y* is a non-negative random variable, to make it compatible with a linear model that has support on ℝ, we construct the GLM using the log-link:


(5) $$\begin{array}{l} \log\left(\frac{\alpha }{\beta }\right)=R{\left(\theta \right)}^{⊤}w≜L\left(w;\theta \right). \end{array}$$

Therefore, we model the conditional distribution as


(6) $$\begin{array}{l} f_{Y|\Theta }\left(y|\theta ;\alpha ,w\right)=\frac{{\left(\alpha e^{-L\left(w;\theta \right)}\right)}^{\alpha }}{\Gamma \left(\alpha \right)}y^{\alpha -1}e^{-\left(\alpha e^{-L\left(w;\theta \right)}\right)y}, \end{array}$$

by rearranging Equation (5) and substituting it into Equation (3).

#### 2.2.2 Fourier basis of regressors

To construct the regressors, *R*(Θ), in our GLM we consider a Fourier basis of regressors since they form an orthogonal basis over [−π, π). This can be formulated as follows:


$$\begin{array}{l} R{\left(\Theta \right)}^{⊤}=[1\;\cos(2\pi \theta )\;\sin(2\pi \theta )\;\cos(2\pi 2\theta )\;\sin(2\pi 2\theta )\ldots \\ \;\cos(2\pi K\theta )\;\sin(2\pi K\theta )], \end{array}$$

where we choose an order *K* to represent the number of Fourier basis pairs we use to fit the conditional expectation.

#### 2.2.3 Model fitting

Define


(7) $$\begin{array}{l} NLL\left(\alpha ,w\right)≜-\overset{T-1}{\underset{t=0}{\sum }}\log\left(f_{Y|\Theta }\left(y_{t}|\theta_{t};\alpha ,w\right)\right)\\ =\overset{T-1}{\underset{t=0}{\sum }}\log\Gamma \left(\alpha \right)-\left(\alpha -1\right)\log y_{t}+\alpha y_{t}e^{-L\left(w;\theta_{t}\right)}\\ +\alpha L\left(w;\theta_{t}\right)-\alpha \log\alpha \end{array}$$

to be the negative log-likelihood, where *t* = 0, …, *T* − 1 is the time index of the time series sample of the amplitude, *y*_*t*_, and phase, θ_*t*_, and *T* is the total number of samples in the data. To fit both α and *w*, we take a two-step approach where we first fit *w* and then use the optimal estimate of *w* to generate an estimate for α. This is because the estimation of *w* is invariant to the value of α, since algebraic manipulation of (7) leads to


(8) $$\begin{array}{l} ŵ\left[\alpha \right]=\arg\underset{w}{\min}NLL\left(\alpha ,w\right)=\arg\underset{w}{\min}\overset{T-1}{\underset{t=0}{\sum }}y_{t}e^{-L\left(w;\theta_{t}\right)}+L\left(w;\theta_{t}\right). \end{array}$$

This minimization problem is convex in *w*, which allows for us to use common convex optimization solvers in order to easily solve for the maximum likelihood estimate of *w*. Now define *L*_*t*_ ≜ *L*(ŵ; θ_*t*_). The solution for $\hat{\alpha }$ is given by


(9) $$\begin{array}{l} \hat{\alpha }\left[ŵ\right]=\arg\underset{\alpha }{\min}\overset{T-1}{\underset{t=0}{\sum }}\log\Gamma \left(\alpha \right)-\left(\alpha -1\right)\log y_{t}+\alpha y_{t}e^{-L_{t}}+\alpha L_{t}-\alpha \log\alpha , \end{array}$$

which is also a convex problem. Our conditional distribution can then be written in closed form by plugging in ŵ and $\hat{\alpha }$ into Equation (6). Note that reliability in estimates of the model parameters increases with sample size. So long as one samples above the Nyquist frequency of the high frequency signal, then even over only one cycle of phase of the low frequency signal, numerous samples of the high frequency signal should be obtained at approximately the same low frequency phase, resulting in reliable estimates.

#### 2.2.4 Model selection

Model selection of the number of Fourier bases, *K*, was performed by using the Minimum Description Length (MDL) principle (Barron et al., 1998). That is, we now let our model be parameterized by K∈K, where $K\subset {\mathbb{Z}}^{+}$ is the set of possible numbers of Fourier bases we can use in the model. We then construct the penalized normalized negative log-likelihood (PNNLL):


$$\begin{array}{l} PNNLL\left(K\right)=\frac{1}{T}NLL\left(\hat{\alpha },ŵ;K\right)+\frac{1}{T}\frac{2K+1}{2}\log\left(T\right). \end{array}$$

Our model order is then given by


$$\begin{array}{l} \hat{K}=\arg\underset{K\in K}{\min}PNNLL\left(K\right). \end{array}$$

### 2.3 Mutual information calculation

We first define the relative entropy between the posterior (prior) distributions *P*_Θ|*Y* = *y*_ (*P*_Θ_) with density *f*_Θ|*Y*_(·|*y*) (*f*_Θ_), respectively, as:


(10) $$\begin{array}{l} D\left(P_{\Theta |Y=y}||P_{\Theta }\right)=\int_{\theta }f_{\Theta |Y}\left(\theta |y\right)\log\left(\frac{f_{\Theta |Y}\left(\theta |y\right)}{f_{\Theta }\left(\theta \right)}\right)d\theta . \end{array}$$

We define our new measure of PAC as the mutual information, *I*, between *Y* and Θ, which can be equivalently be defined as Gray (2011):


$$\begin{array}{l} I\left(Y;\Theta \right)={𝔼}_{Y}\left[D\left(P_{\Theta |Y=y}||P_{\Theta }\right)\right]. \end{array}$$

We use this specific expression for the mutual information to take advantage of the fact that the relative entropy, given by (Equation 10), can be computed by an integral over finite support [−π, π) which enables a provably good approximation by Riemann sum. We note that the mutual information's increase away from zero can be interpreted as a degree of increase in PAC.

To calculate Equation (10), we can utilize Bayes' rule to find the posterior density on phase:


$$\begin{array}{l} f_{\Theta |Y}(\theta |y)=\frac{f_{Y|\Theta }(y|\theta )f_{\Theta }(\theta )}{\int_{\theta^{\prime }}f_{Y|\Theta }(y|\theta^{\prime })f_{\Theta }(\theta^{\prime })d\theta^{\prime }}, \end{array}$$

where *f*_Θ_(θ) is given by Equation (1) and *f_Y_*_|Θ_(*y*|θ) is given by Equation (6) after model fitting.

### 2.4 Information density as a time-resolved measure of PAC

Previous work done by Martinez-Cancino et al. (2019), shows the potential for the information density (“local” mutual information) as a measure for PAC defined at every moment in time. In their work they use a nonparametric estimator of mutual information (KSG estimator), whereas here we propose to use our parametric model to estimate the same time-varying quantities without bias. We first define the information density as Gray (2011)


(11) $$\begin{array}{l} i\left(y,\theta \right)=\log\left(\frac{f_{Y,\Theta }\left(y,\theta \right)}{f_{Y}\left(y\right)f_{\Theta }\left(\theta \right)}\right)\\ =\log\left(\frac{f_{Y|\Theta }\left(y|\theta \right)}{f_{Y}\left(y\right)}\right), \end{array}$$

where the lowercase *y* and θ represent specific observations of the random variables *Y* and Θ. We can interpret this as the relative reduction in surprise we have about any particular *y* given that an observation of θ. In other words, this quantity is giving a measure of how much of an improvement we can predict a specific outcome *y* by also having an observation of a specific outcome θ.

Given that we fit a joint distribution over the data *Y*_*t*_ and Θ_*t*_, we can then evaluate Equation (11) at every time point for the specific data that we have. We can write this as


$$\begin{array}{l} \text{idPAC}\left(t\right)=i\left(y_{t},\theta_{t}\right)=\log\left(\frac{f_{Y|\Theta }\left(y_{t}|\theta_{t}\right)}{f_{Y}\left(y_{t}\right)}\right), \end{array}$$

for every sample pair (*y*_*t*_, θ_*t*_). We call this the information density PAC, or idPAC for short.

### 2.5 Goodness-of-fit assessment

We assess the goodness-of-fit (GOF) of our model by considering how well the data are described by their conditional distributions fit by the GLM in Equation (6). This assessment is crucial, since the measures obtained from the model, e.g., mutual information, only describe the data to the extent that the model fits the data. We do this by utilizing the so called probability integral transform (PIT), otherwise known as the universality of the uniform. The PIT states that if one inputs any random variable *X* into its own CDF, then the new random variable *U* = *F*_*X*_(*X*) will be uniformly distributed on [0, 1]. In the GLM case, we plug in the data *Y*_*t*_ into its conditional CDF, which can be calculated from Equation (6) with optimized parameters given by Equations (8, 9). This is given by


$$\begin{array}{l} U_{t}=F_{Y|\Theta }\left(Y_{t}|\Theta_{t};\hat{\alpha },ŵ\right), \end{array}$$

where we can find the CDF by integrating the PDF in Equation (6). Note, we use the conditional CDF as opposed to the marginal CDF, *F*_*Y*_(*y*), because traditional GOF assessment requires identically and independently distributed (i.i.d.) data. Although each individual *Y*_*t*_'s statistics may vary with *t*, our model assumes that the conditional distribution of *Y*_*t*_ given Θ_*t*_ is invariant to t, and thus we use the conditional CDF approach.

Then we construct the empirical CDF of the *U*_*t*_ and compare it to the CDF of a theoretical uniform random variable on [0, 1] which we will call *F*(*U*). The empirical CDF is given by


(12) $$\begin{array}{l} \hat{F}\left(u\right)=\frac{1}{T}\overset{T-1}{\underset{t=0}{\sum }}1_{\left\{U_{t}\leq u\right\}}. \end{array}$$

If the model does indeed fit the data well then the empirical CDF and the true CDF should match up well. Note that **1**_{*U*_*t*_ ≤ *u*}_ is the indicator random variable and evaluates to 1 if *U*_*t*_ ≤ *u* and 0 otherwise. Equation (12) is equivalent to counting the number of *U*_*t*_ ≤ *u* for any chosen value of *u* and dividing by the total number of samples.

To test our model fit we use mouse iEEG data provided by Scheffer-Teixeira et al. (2011). There are two datasets provided in their work: the first of which investigates high-gamma (HG, 60–100 Hz) and theta band (5–10 Hz) coupling; the second of which investigates high frequency oscillation (HFO, 120–160 Hz) and theta band coupling. Each dataset is 300 s long. We fit our model to all 300 non-overlapping one second segments in each dataset. These results are reported as probability plots, which are parametric plots with the empirical CDF of *U*, $\hat{F}\left(u\right)$, on the y-axis and the CDF of a uniform distribution, *F*_*U*_(*u*) = *u*, on the *x*-axis.

### 2.6 Corrections for multiple comparisons

Often times in neuroscience, one is presented with the problem of simultaneously testing multiple hypotheses. This is known as the multiple hypothesis testing (or multiple comparisons) problem. For example, in the case of PAC, one may be testing to see if any of *N* low frequencies are coupled to any of *M* high frequencies. Since the collected data is random, any functions of the data (e.g. proposed measures described in this manuscript), are also random variables. In the classical setting, one would generate surrogate data, by permuting the dataset, which should destroy PAC structure, to compute the PAC metric under the null hypothesis. By computing statistics on this after multiple generations of surrogate data, a null distribution can be calculated to then determine the probability of the PAC metric or anything more extreme occurring under the null hypothesis. Finally, one may compare this to a threshold, say α = 0.05, and declare significance if the *p*-value < α.

Since multiple hypothesis tests are implemented, and α is the probability of rejecting the null hypothesis when the data was drawn from the null distribution, if we then compare all of our tests to the α = 0.05, then ~5% of all tests on null data will generate a false positive. To combat this, we turn to multiple hypothesis testing techniques that control the false discovery rate (FDR), defined below


$$\begin{array}{l} \text{FDR}=𝔼\left[\frac{\text{\# false positives}}{\text{\# false positives + \# true positives}}\right]. \end{array}$$

Control of this quantity is much like in the single hypothesis test case, where we might set α = 0.05, such that, FDR < α. In this case, instead of 5% of tests associated with null data generating a false positive, 5% of all results we declare positive will be false positives. Unlike the single hypothesis testing case, the significance threshold for each individual test becomes more strict.

#### 2.6.1 Benjamini-Hochberg procedure

To control the false discovery rate, we use the well known Benjamini-Hochberg (BH) Procedure (Hochberg and Benjamini, 1990) which guarantees that the expected value of the ratio false positives to all positives is at most α. Note, that this guarantee requires an assumption on the independence/positive dependence of the *p*-values. This procedure is implemented by computing all of the individual *p*-values for each of the hypothesis tests and then sort them from low to high. Assuming *N* such tests, the line $y=\alpha *\frac{i}{N}$, where *i* = 1, …, *N* is created after sorting the index of each test in an increasing manner. The critical value is then calculated as


$$\begin{array}{l} c=\arg\underset{i}{\max}p\left(i\right)<\alpha *\frac{i}{N}, \end{array}$$

where *p*(*i*) is the *i*th smallest *p*-value. Note that there may be *p*-values for indices below *c* that are above the test line that are declared positive. This is a function of the procedure and it still controls the FDR at rate α. Note that one can alternatively adjust all of the sorted *p*-values by computing $p\left(i\right)*\frac{N}{i}$ and comparing the adjusted *p*-values to the horizontal line at α. This is what we will show in this paper for the experiments where we have a multiple testing problem, since it is easier to visualize than using raw *p*-values. Note, the sorted *p*-values after adjustment may not increase monotonically, but will yield the same results.

Since the guarantees on this procedure rely on the independence/positive dependence on *p*-values, one may also choose to use a modified BH procedure, known as the Benjamini-Yekutieli procedure, which makes this procedure more strict by lowering the threshold by a constant factor (Benjamini and Yekutieli, 2001).

### 2.7 Experiment

#### 2.7.1 Synthetic data

In order to assess the ability of cross frequency coupling (CFC) methods to correctly identify PAC in signals, we generated instances of phase-amplitude coupled synthetic signals. The generated signals *x*(*t*) were inspired by Tort et al. (2010) and are defined as follows:


(13) $$\begin{array}{l} x\left(t\right)=A_{f}\left(t\right)\sin\left(2\pi f_{high}t\right)+A_{s}\sin\left(2\pi f_{low}t\right)+\eta \left(t\right), \end{array}$$

where *f*_*high*_ is the frequency of the fast signal, *f*_*low*_ is the frequency of the slow signal, *A*_*f*_(*t*) is the modulated amplitude, and $\eta \left(t\right)\sim N\left(0,\sigma^{2}\right)$ is Gaussian noise. The modulated amplitude is:


(14) $$\begin{array}{l} A_{f}\left(t\right)=\frac{\chi \sin\left(2\pi f_{low}t\right)+2-\chi }{2}, \end{array}$$

where χ ∈ [0, 1] is the coupling coefficient. Notice that larger values of χ allow the slow sinusoid to more heavily affect the amplitude of the fast signal.

#### 2.7.2 Comparison to current methods

To test the discriminatory ability of our PAC method, we generated a synthetic data set with parameters chosen to mimic the gastric slow wave recorded by electrogastrography (EGG) and the alpha band activity in the brain. We generated both coupled and uncoupled signals with varying coupling coefficients. We generated the dataset with the specifications in Table 1.

**Table 1:** Signal parameters.

*f*_*high*_	10 Hz	*f*_*low*_	0.05 Hz
*f*_*s*_	50 Hz	χ	[0, 0.3]
*A*_*s*_	1	SNR	0 dB

The noise variance, σ^2^, was set to make the synthetic signal's signal-to-ratio (SNR) 0 dB to simulate low SNR signal recordings. *A*_*s*_ is chosen as 1 for simplicity. We chose signal lengths of 20 s to mimic one cycle of the gastric slow wave to compare how our methods perform in time-resolved settings. The sampling rate *f*_*s*_ was chosen to be high enough to be above the Nyquist rate, but also low enough to reduce computational time.

We use a 4th order zero-phase Butterworth filter on our synthetic signal, *x*(*t*), on two separate passbands around *f*_*high*_ ([8, 12] Hz) and *f*_*low*_ ([0.03, 0.07] Hz), to obtain our respective fast and slow signals, *x*_*f*_(*t*) and *x*_*s*_(*t*). We extract *A*_*f*_(*t*) and ϕ_*s*_(*t*) as described in Section 2.1.2.

We also compare our method to existing methods of CFC: phase-locking value (PLV), mean vector length (MVL), and the Modulation Index (MI). These are some of the most commonly implemented methods of CFC analysis. They are defined as follows:


(15) $$\begin{array}{c} \text{PLV}=\frac{1}{T}|\overset{T-1}{\underset{t=0}{\sum }}e^{i\left(\phi_{1}\left(t\right)-\phi_{2}\left(t\right)\right)}|, \end{array}$$

(16) $$\begin{array}{c} \text{MVL}=\frac{1}{T}|\overset{T-1}{\underset{t=0}{\sum }}a_{1}\left(t\right)e^{i\left(\phi_{2}\left(t\right)\right)}|, \end{array}$$

(17) $$\begin{array}{c} \text{ndPAC}=\frac{1}{T}|\overset{T-1}{\underset{t=0}{\sum }}{ã}_{1}\left(t\right)e^{i\left(\phi_{2}\left(t\right)\right)}|,\text{and} \end{array}$$

$$\begin{array}{c} \text{MI}=\frac{D\left(P||U\right)}{\log\left(N\right)}, \end{array}$$

(Tort et al., 2010; Özkurt, 2012; Hülsemann et al., 2019). For Equations (15, 16, 17), the variables *a* and ϕ are subscripted by a number to denote two different signals. In Equation (17) ã_1_(*t*) refers to the normalized amplitude time series (mean subtracted and variance made unity). *D*(*U*||*P*) is the relative entropy of the conditional expectation, **E**[*Y*|Θ], binned by phase and normalized to a distribution, *P*, with respect to the uniform distribution, *U*, where *N* is the number of bins in the probability mass function (PMF) of *U* and *P*. Note that PLV (15) does not take into account amplitude information and is a phase-phase coupling method. This allows us to have a negative control which should not be able to distinguish between phase-amplitude coupled signals.

For each experiment, we generate a dataset of 50 uncoupled signals (χ = 0) and 50 coupled signals (0 < χ < 1) and generate a receiver operating characteristic (ROC) curve with corresponding area under the curve (AUC)—see **Figure 3**. We choose K={1,2,3,4,5}. We then run 100 trials of each experiment with the same coupling coefficient to generate a confidence interval for each method.

#### 2.7.3 Time-resolved measurements of PAC

In order to test the validity of our idPAC measure we construct synthetic signals similar to those in Equations (13, 14), except we let the coupling coefficient χ be a function of time. That is, we construct χ(*t*) to be either a square wave or ramp function to test our algorithm's ability to capture the transient PAC phenomena. As reported before in the seminal work by Martinez-Cancino et al. (2019), the frequency of the phase signal tends to create artifacts in the idPAC estimates. Therefore, we borrow from their work and choose to lowpass filter the idPAC to below the frequency of the phase signal. The idPAC also has the potential to produce negative values, unlike the mutual information, so we also choose to set any value of the idPAC < 0 to be equal to 0. We also mirror former work and set the SNR = 10 dB to validate our method.

#### 2.7.4 Event-related phase-amplitude coupling

In this work, we also showcase the ability of our method to generate event-related PAC, as first demonstrated by Voytek et al. (2013). An ERP is traditionally a characteristic short-time transient electrophysiological response to a stimulus of some variety (visual, auditory, etc.). Many trials of the same stimulus are given to a subject to collect enough data to observe clear a ERP through noise by averaging the trials together. Event-related PAC utilizes the many trial paradigm of event-related potentials (ERPs) in order to obtain an estimate of phase-amplitude coupling at each time point in the overall ERP. To estimate PAC at every time point, we utilize our time-resolved measure of PAC (information density). Specifically, we organize our electrophysiology data into matrices that are of shape number of trials × number of time points. Then we calculate the phase matrix of the low frequency signal of interest and the amplitude matrix of the high frequency signal—both of same shape as the data, and filtering as needed. At each time point we then collect a full cycle's worth of phase and amplitude data and compute the information density PAC for each trial, with the time point of interest taken as the midpoint of the cycle. We define a full cycle to be one period of the oscillation of the lower frequency signal, in this case, the phase. We do this in order to ensure that we sample the full range of phase values. To illustrate more clearly, take *T* = 101 samples to be a full cycle, and assume we are interested in the time point *t* = 1,000 samples. To estimate the ERPAC at time *t*, we would take all data in the phase and amplitude matrices associated with *t* = 950 to *t* = 1,050 for all trials, and then fit our gamma GLM. Then, we would compute the idpac for all trials at the datapoints associated with *t* = 1,000. Note that the output of each of this computation is a single vector of length number of trials associated with the time point of interest. To obtain the time series of ERPAC per trial, we then perform this procedure for every point in the data matrix. Note that for points near the beginning and end times of the matrices, one will have to append a half cycles worth of data to be able to perform the procedure at those time points. After we obtain the ERPAC time series per trial, we then address the frequency leakage problem by lowpass filtering at the center frequency of the phase signal and truncating the lower end of the output at zero for every trial individually. To obtain the mean event-related PAC (ERPAC) for all of the trials we take an average of the idPAC over the trial axis.

To demonstrate this, we generate data as in the previous section for time-resolved PAC, however, in this case we generate 100 trials of data. The data taken in real ERP settings also usually experiences jitter in the stimulus and natural variation in the brain response timings, so we uniformly choose a random jitter of 1–100 samples for each trial. We choose to couple signals at 0.05 and 10 Hz as in our ROC curve experiment. The sampling frequency here was chosen to be 50 Hz to speed up computational time.

### 2.8 Demonstration in electrophysiological data

We demonstrate the potential of our method in one-shot, time-varying, and ERP analyses in electrophysiological data. Specifically we validate using mouse intracranial EEG (iEEG) from a previous study by Scheffer-Teixeira et al. (2011), with previously validated PAC findings and compare their findings to findings using our method. To evaluate the potential of our method in time-varying cases we used sleep spindle data from the YASA Git repository and mismatch negativity (MMN) data previously collected by our lab (Vallat and Walker, 2021). In both cases we demonstrate the information density PAC evaluated on single spindles or ERP trials, and the ERPAC that is fit using many trials at once.

#### 2.8.1 Mouse intracranial EEG data

##### 2.8.1.1 Detection of oscillations in mouse iEEG Data

After evaluation of the method on synthetic data we also wanted to test our algorithm's performance on electrophysiologic recordings. We use the dataset provided in Scheffer-Teixeira et al. (2011), which calculates information transfer from the medial entorhinal cortex to the CA1 region of the hippocampus in rats and evaluates PAC using the Modulation Index. The rats were implanted with multi-electrode microwire arrays, which allows for recording of intracranial EEG data, and anesthetized with both ketamine and xylazine. This data has high signal-to-noise ratio can thus can serve as a reference to compare our technique to the modulation index and replicate past findings in electrophysiologic data. The dataset is the same as the one used for GOF analysis in Section 2.5. It consists of two separate 300 s recordings sampled at 1 kHz for different couplings: HG (60–100 Hz) to theta band (5–50 Hz) and HFO (120–160 Hz) to theta band.

##### 2.8.1.2 Comodulogram

To assess PAC in the data we build what is called a comodulogram, which allows one to scan over many different frequency bands for the fast and slow signals to do exploratory analysis on data to find PAC. This is the most common way of reporting PAC findings in the literature. To calculate the comodulogram, we first note that we filter one channel of the iEEG data into low and high frequency bands. For the low frequency bands we scan over 4 Hz frequency bands starting from 0.5 and ending at 20.5 Hz with shifts of 2 Hz to generate $S_{low}=\left\{x_{l,1}\left(t\right),x_{l,2}\left(t\right),\ldots ,x_{l,m}\left(t\right)\right\}$, which is the set of *m* low frequency signals. We do this process analogously for the high frequency signals in 10 Hz bandwidths with shifts of 5 Hz from 0.5 Hz to *f*_*upper*_ Hz to generate $S_{high}=\left\{x_{h,1}\left(t\right),x_{h,2}\left(t\right),\ldots ,x_{h,n}\left(t\right)\right\}$, the set of *n* high frequency signals. Unlike in the low frequency case, to determine the upper bound for the frequency range (*f*_*upper*_) we will search over, we employ a technique developed by Donoghue et al. (2020): *fitting oscillations & one over f* (FOOOF). This technique allows us to detect the presence of oscillatory peaks in the power spectra of the data, which allows us to separate real oscillations from Gaussian and $\frac{1}{f}$ noise. We use this to detect the highest frequency peak present in the data and use its center frequency plus half of its bandwidth as the upper limit over which we scan. The parameters of the FOOOF algorithm for the HG-theta data are: peak_width_limits=[1, 20], min_peak_height = 0.15. The parameters for the HFO-theta data are: peak_width_limits = [1, 25], min_peak_height = 0.2. Both datasets had a frequency range from [0, 200] Hz to scan over, and the inputs to the FOOOF algorithm were Welch periodograms with a Hann window of size 1,024 samples and an overlap of 512 samples.

After the filtering and identification procedure, we then calculate


$$\begin{array}{c} \text{PAC}\left(i,j\right)=M\left(x_{l,i}\left(t\right),x_{h,j}\left(t\right)\right), \end{array}$$

where $ℳ:S_{low}\times S_{high}\to \mathbb{R}$ represents a function to calculate some PAC measure (ours or Modulation Index) calculated between two input signals. PAC(*i, j*) is then plotted and reported as a heatmap for the real iEEG data—see **Figure 6**. We perform both the BH procedure to correct for the multiple testing problem here. We compute *p*-values by first generating 100 surrogate comodulograms to approximate a null distribution for each PAC(*i, j*). Only 100 surrogates were used here because of limited computational resources. Each surrogate was computed by shifting the phase of the dataset—for each *i, j* pair—by a random amount of at least 1 second. This was to ensure that the phase still that of an oscillator as opposed to a full permutation which would have destroyed all structure. We then fit a gamma distribution to each of the 100 surrogate PAC(*i, j*)s, and compute the *p*-value as the probability of observing a our actual PAC value under the null hypothesis. We performed a Kolmogorov–Smirnov test between the surrogate distribution and the fit gamma distribution to ensure that the approximation was reasonable. On each of the comodulograms, only the PAC(*i, j*) that were deemed significant are shown. All other values are set to zero in the heatmap.

#### 2.8.2 Joint high-density EEG and EGG topoplots

Resting-state simultaneous EEG and EGG data was used to evaluate the ability of our PAC measure to recreate results from Richter et al. (2017) that demonstrated the existence of electrophysiological gut-brain PAC. The data were collected under Stanford IRB 68900. We recorded high-density EEG data at 1 kHz using a 128-channel EEG system from Electrical Geodesics. We also collected EGG data at 250 Hz using an 8-channel OpenBCI Cyton Board. Ten minutes of resting-state data was used for analysis. To recreate these results we chose to look at the coupling of the electrogastrogram with the alpha band signal (8–12 Hz) of the EEG. We synchronized both recordings with two experimenters starting the recording simultaneously on separate devices. We approximate the synchronization error to be on the order of 0.5 s, which is significantly less than the period of 20s pertaining to the gastric slow wave, suggesting a minor effect on stomach brain coupling measures.

To remove artifacts, we preprocessed both the EEG and EGG data using respective preprocessing pipelines. First, both datasets were decimated to 125 Hz sampling rates to decrease the amount of data and so that they could be paired point-wise in time. The EEG data was then bandpass filtered to [1, 50] Hz using a zero-phase 4th order Butterworth filter to remove drift and 60 Hz line noise before artifact rejection. The MNE python package was then used to perform electrooculogram (EOG) regression on the data to remove eye blink and movement artifacts. At this point, the EEG data were then filtered to the alpha band frequency, again using a zero-phase 8th order Butterworth filter (implemented through a forward and backward pass of a 4th order filter). For the EGG data, a local adaptive wiener filter was used to remove motion artifacts as in Gharibans et al. (2018). The EGG data was then filtered to [0.03, 0.07] Hz frequency band using a 8th order zero-phase Butterworth filter (also by forward-backward filtering).

The “best” EGG channel was selected by first referencing the EGG channels to all other channels (i.e., compute all pairs *x*_*i*_(*t*)−*x*_*j*_(*t*)) and then computing the power around the gastric slow wave frequency band. The referenced EGG channel with the highest power in the EGG frequency band was then chosen for analysis.

We then generated topographic plots of PAC between the chosen EGG channel and all 128 EEG channels. Both the modulation index and our PAC measure were computed between the chosen EGG channel and all EEG channels separtely. Statistical significance was evaluated through surrogate data analysis. Surrogate data were generated by randomly shifting the phase of the EGG signal in time and then recomputing the corresponding PAC values. A total of *N* = 5,000 surrogates were generated for each subject. Assuming that the EGG and EEG signal are coupled at specific instances in time, this process allows us to disrupt that coupling and generate samples of PAC values from the null distribution. For each iteration of surrogate data, the EGG signal was shifted by a random amount of at least 60 s to ensure disruption of this coupling. This process, in contrast to surrogate analysis where the phases are permuted, allows us to keep the shifted EGG signal as a physically relevant oscillator. The *z*-scores for each PAC measure were computed using their respective surrogate null distributions, and the plotted as a heat map over a scalp model using the MNE Python package—see **Figure 7**. Note, that null distributions were generated separately for each EEG channel. *p*-values were computed empirically from the surrogate samples by computing the proportion of surrogate data greater than or equal to the actual PAC measures. Corrections for multiple comparisons are shown for both the BH procedure—see **Figure 7**.

#### 2.8.3 Sleep spindle electroencephalography data

It has long been know that sleep spindles have thalamic generators and occur during slow wave sleep, when slow wave electrophysiologic oscillations are present (Destexhe and Sejnowski, 2009). It has also been proposed that there may be a mechanism of cortical to thalamic control of sleep spindle generation, where slow wave oscillations may be one of the culprits that organize these sleep spindles. Thus it is an interesting question to understand, if and how, phase-amplitude coupling between slow waves and faster oscillating sleep spindles may arise. Polysomnography (PSG) data for use in analysis of sleep EEG was obtained from the YASA Git repository – an open source Python package for automated sleep staging of PSG data (Vallat and Walker, 2021). Data used to train and test YASA was obtained by Vallat et al., from the National Sleep Research Repository (NSSR) and the Dreem open dataset (Dean et al., 2016; Guillot et al., 2020).

Initial preprocessing of the data was done first by utilizing the YASA package to do sleep staging on the Cz electrode of the PSG. N1, N2, and N3 stages of sleep were identified, and then we further utilized YASA to identify sleep spindles in the data. Each of the sleep spindles were collected by identifying the peaks of each sleep spindle (also done using YASA) and then collecting 2 s of data before and after the peaks of the spindles. All spindles were then aligned against each other in an *N* × *T* matrix, where *N* is the number of sleep spindles and *T* is the time window collected around each sleep spindle. Any sleep spindles with peaks within 3 s of each other were excluded from the downstream analysis in order to prevent any possible leakage of one sleep spindle into the time window of another sleep spindle.

We then analyzed the time-varying PAC in the sleep spindles using both the idPAC and ERPAC methodologies. The frequency bands of choice for analyzing sleep spindles using PAC were the slow oscillation (SO, 0.1–1.25 Hz) and sigma band (12–16 Hz). This follows the work done by Winer et al., where they show that Tau and β-Amyloid burden was negatively correlated with SO-sleep spindle coupling (Winer et al., 2019). In the idPAC paradigm, we filter each sleep spindle into the corresponding low and high frequency bands and then fit our gamma GLM on each individual sleep spindle and calculate the information density at each time point. In the ERPAC paradigm, we first take our phase and amplitude data matrices as specified in the Methods section above and then mirror the first and last 0.5 s of data and reflect them about the beginning and end time points. This way we can still generate estimates of the ERPAC phenomenon for the whole length of time of each spindle. Alternatively, one can choose to take their trial epochs to be of longer length and “sacrifice” the edge data, however, here we choose to reflect data to mimic a limited data scenario (e.g., experiments for which there is very limited time between trials). The analysis of ERPAC is then exactly as mentioned in the ERPAC setup in the Methods section. The ERPAC trace for the whole dataset was reported as a single trace and the idPAC associated with each time point was reported in a heatmap.

#### 2.8.4 Mismatch negativity event-related potentials

The mismatch negativity data was collected at UCSD in concordance with IRB number 130484. Subjects in this dataset were given auditory stimuli and were subjected to an oddball paradigm where they were given one tone to listen to every 500 ms and then periodically given an oddball tone that elicited an ERP. Data from one experiment using the Fp2 electrode was used in the analysis for this study. The data were epoched around the oddball trials with 200 ms pre-stimulus and 800 ms post-stimulus and aligned using the stimulus timings. The low frequency band was filtered to the delta band (0.5–4.0 Hz) and the high frequency band was filtered to the ERP frequency (8.0–15.0 Hz). We then performed both the idPAC analysis on each ERP trial and also reported the average across trials. For the ERPAC analysis, as in the sleep spindle data, the first and last 0.5 s of the data were reflected about the beginning and end time points to pad the data. Mirroring the sleep spindle analysis, the ERPAC trace for the whole MMN dataset was reported as a single trace and the idPAC associated with each time point was reported in a heatmap.

## 3 Results

### 3.1 Goodness-of-fit

**Figure 2:**
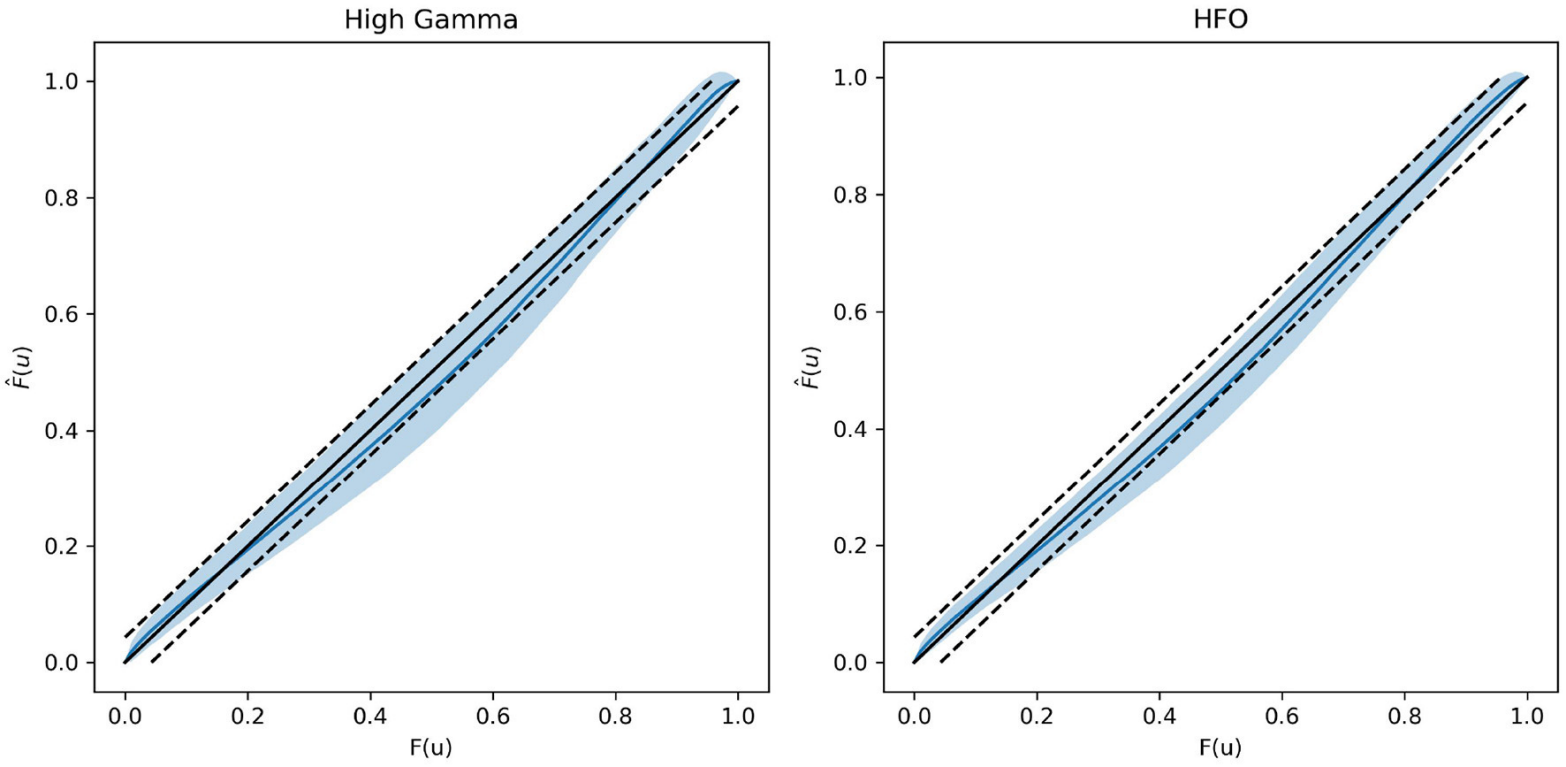
Probability plots showing exemplary goodness-of-fit in the real neural data from Scheffer-Teixeira et al. The left plot represents data associated with HG and theta band PAC. The right plot represents data associated with HFO and theta band PAC. Data were fit over consecutive non-overlapping 1 second chunks. The central solid black 45 degree line represents perfect model fit, and the blue curve represents the mean model fit. The dashed black lines represent the 95% confidence interval for the data being generated by the model, and the blue shaded region represents 95% interval of model fits. Larger distances from the 45 degree line represent worse model fit and vice versa.

In Figure 2, we plot probability plots to demonstrate the GOF in real neural data. We use the data provided in the Scheiffer-Texeira et al. study that investigated theta band activity coupled to both high gamma band (HG, 60–100 Hz) and high frequency oscillations (HFO, 120–160 Hz) and plot as described in the Methods section above. We see that over all chosen one-second segments of data, the model fits very well in the plots shown in Figure 2, where perfect fit would correspond to the blue curve exactly on the 45 degreee line. The dashed lines represent 95% confidence intervals for the data actually coming from the model and the blue shaded region is the 95% interval of model fits. We see that for all data, the probability plots stay mostly within our confidence intervals, with a modest deviation from the dashed line (which is expected for physiologic data). These results imply that our model fit is sensible and will produce reliable estimates of the mutual information.

### 3.2 ROC analysis on synthetic data

**Figure 3:**
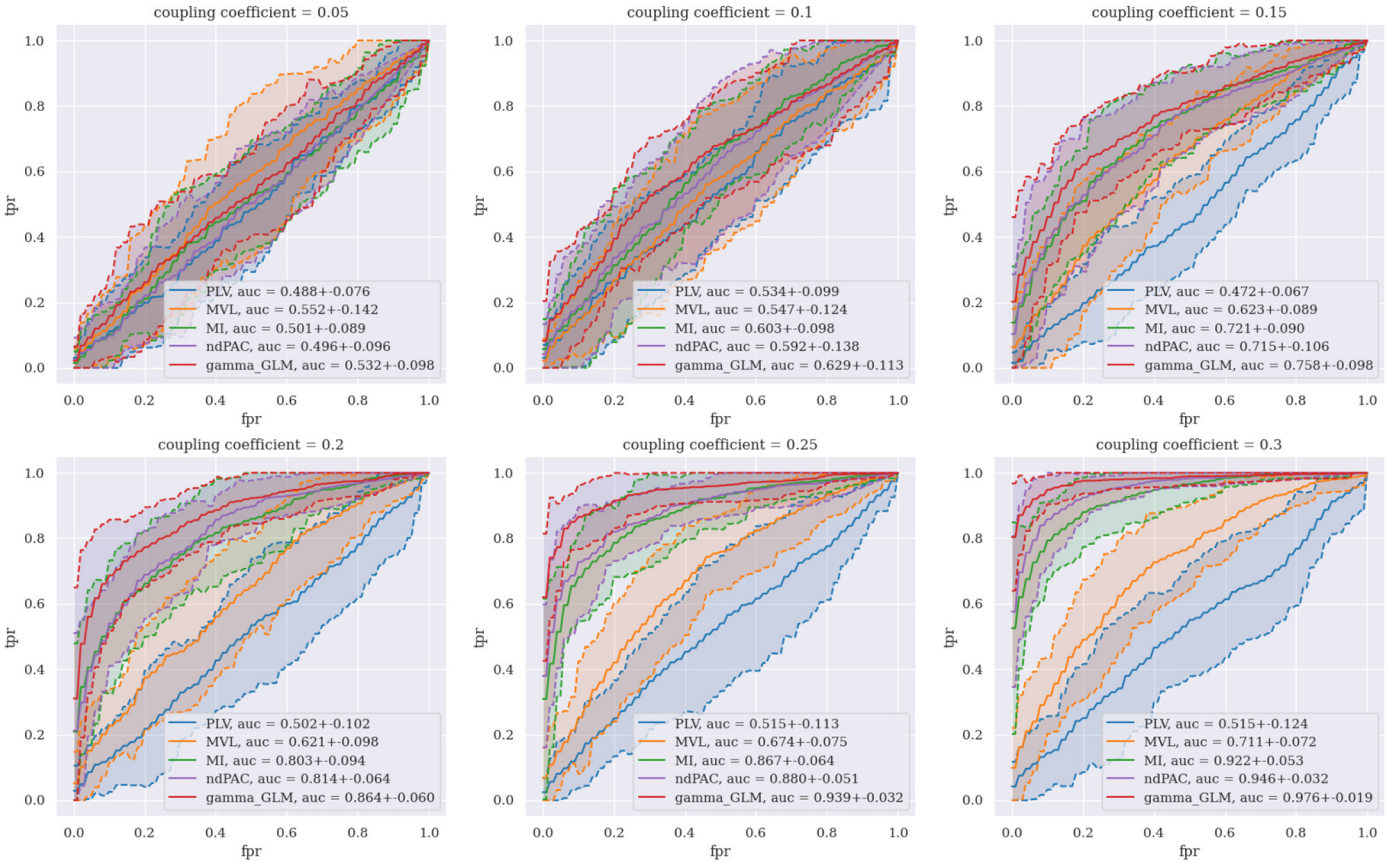
Receiver operating characteristic curve of differing CFC methods across multiple different coupling coefficients.

Figure 3 shows the ROC curves generated for all five of the CFC methods tested over six different coupling coefficients. Each curve was generated using 60 total synthetic data signals (30 coupled and 30 uncoupled), and 95% confidence intervals for the ROC curves were plotted as dashed lines with shading. We use small values of the coupling coefficient to test how the algorithms can pick our coupling in noisy environments. When the coupling coefficient is very large, it becomes very easy to distinguish between PA coupled and uncoupled signals with most PAC algorithms and is therefore not interesting to study. In an ROC curve, we set different thresholds of PAC values to classify uncoupled and coupled signals and then plot the true positive rate (TPR) against the false positive rate (FPR) for each threshold value. Any curve that falls along the 45 degree diagonal line is equivalent to random guessing. Curves closer to the top left corner have higher classification performance since they can achieve higher TPRs at the same FPR as compared to a curve not as close to the top left corner. We quantify this perfomance using the AUC of the ROC curve, which takes on values between 0 and 1. We find that in simulation with a coupling coefficient that is sufficiently small (≤0.1), none of the algorithms are able to distinguish between coupled and uncoupled signals at a rate greater than chance. However for small, but sufficiently large, coupling (0.1 < χ ≤ 0.3), we see that our PAC metric (in red) outperforms all other PAC metrics. We also see that, as expected, the performance of all PAC metrics increases as the coupling coefficient increases with our PAC metric achieving near perfect AUC at a coupling coefficient of 0.3. Note that the PLV (in blue) is a phase-phase coupling metric and serves as a control since it should not be able to pick up PA coupled signals.

### 3.3 Time-resolved PAC experiments

**Figure 4:**
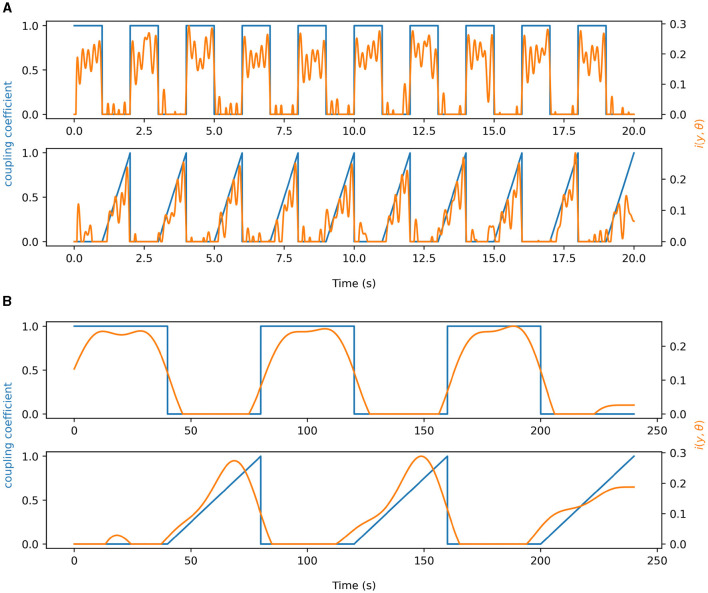
Demonstration of information density as a time-resolved measure of PAC. The blue signals in each plot represent time-varying coupling coefficients associated with synthetic data. The orange curves show the idPAC as a function of time to estimate coupling. **(A)** Time-resolved coupling demonstrated using 5 and 40 Hz signals. **(B)** Time-resolved coupling demonstrated using synthetic data representing the gastric slow wave (0.05 Hz) and alpha band EEG (10Hz) to simulate physiology. Time-varying coupling coefficients for both square waves and ramp functions were well represented by idPAC in both **(A)** and **(B)**.

In Figure 4, simulations for time-varying brain-brain and gut-brain coupling are shown. The blue traces in each figure represent the true underlying coupling coefficient, while the orange traces represent the time-varying estimates of PAC. Both Figures 4A, B we note that for square wave and sawtooth wave simulations of the coupling coefficent, the idPAC was able to reliably estimate the true coupling and react to fast and slow transient changes in the underlying coupling coefficient. Between the brain-brain and gut-brain coupling simulations, we also notice that in the brain-brain case the idPAC was able to track changes in PAC more sharply than in the gut-brain case most likely due to the faster time scales and the more similar orders of magnitude of the frequencies involved (i.e., 5 and 40 Hz vs. 0.05 Hz and 10 Hz). This suggests to us that in understanding PAC for the gut-brain axis, it is imperative to study and validate these phenomena for very low frequency signals.

### 3.4 Event-related PAC simulations

**Figure 5:**
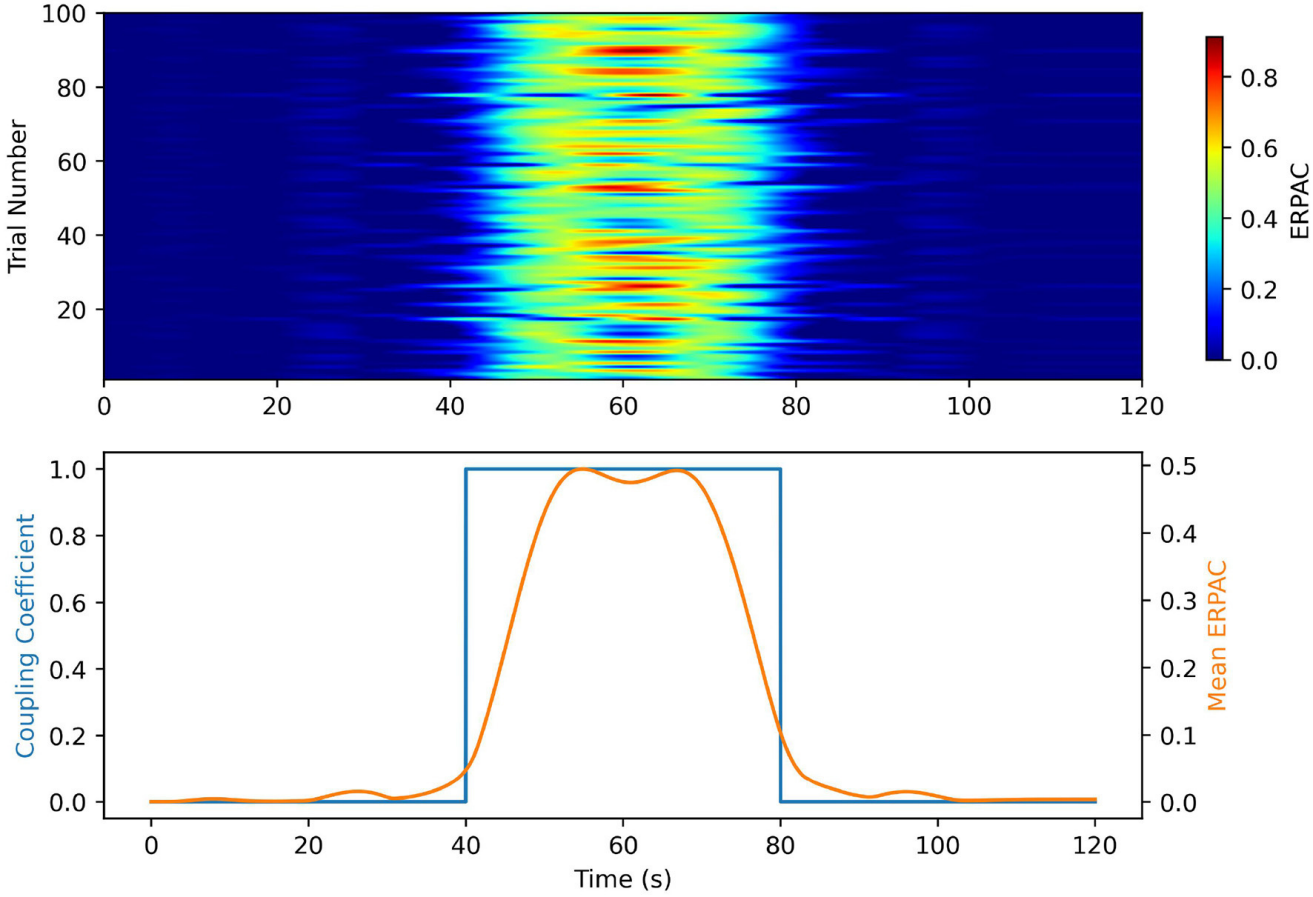
Event Related PAC simulations in the gut-brain frequencies bands. **(Top)** A heatmap of the ERPAC time series by trial. The rows represent trials and the columns represent time points. The color bar on the heatmap represents ERPAC value. Note that the ERPAC value per time series well represents the coupling time series over time, and the changes in onset of coupling are representative of the introduced timing jitter. **(Bottom)** Mean ERPAC time series plotted against the ground truth coupling coefficients. The mean ERPAC time series tracks well with the shape of the coupling coefficient. The inability of the method to follow the sharp changes in coupling is likely due to the introduced jitter and filtering of the ERPAC to eliminate the leakage from the low frequency oscillator.

In our ERPAC experiment, Figure 5 shows that we achieve performance similar to that in the idPAC case. The heatmaps demonstrate that our method was able to find consistent PAC associated with each time point in the simulation. The estimates of ERPAC closer to the edges of the square wave coupling coefficient are less sharp and the possible explanations are two-fold: (1) the very low frequency of the gastric signal makes it difficult to estimate sharp changes in PAC, and (2) the jitter introduced in the timing of the simulated ERPs causes the alignment of the signals to be imperfect outside of the center of the square wave.

### 3.5 Performance in mouse iEEG

**Figure 6:**
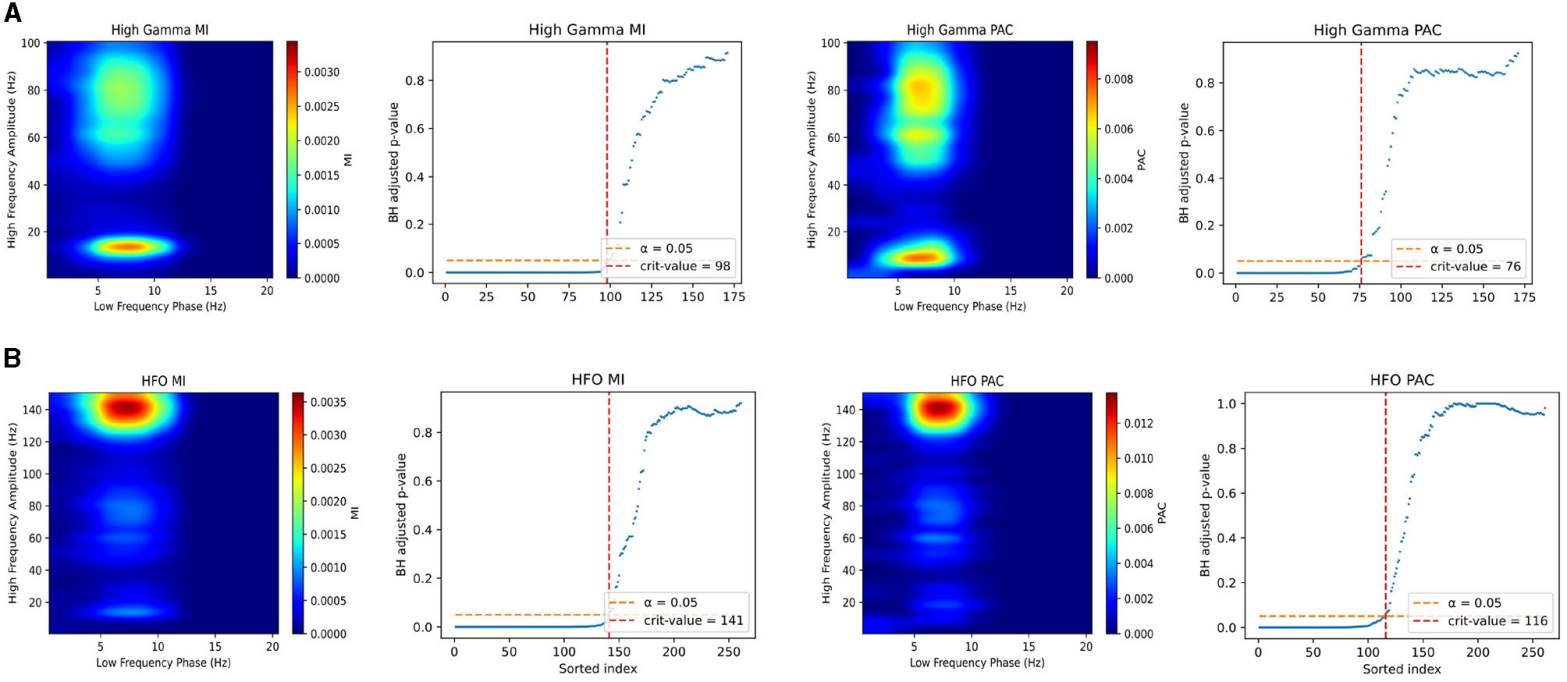
Comparison of Modulation Index and our PAC measure in mouse iEEG data with corrections for multiple comparisons. **(A)** Comodulograms for theta-HG coupling using Modulation Index (left column) and our measure (right column). Next to each comodulogram is te associated multiple comparisons plot, showing the number of significant locations. The x-axis of the plots are sorted using the unadjusted *p*-values from low to high. The dashed orange line is the α = 0.05 threshold for FDR control, and the vertical red dashed line is the critical value for the number of significant tests. **(B)** (left) Comodulograms produced for theta-HFO coupling using Modulation Index (left column) and our measure (right column). This figure plots only MI and PAC values for significant frequency pairs. Note, our method, after correction for multiple comparisons, results in less “leakage”.

Our results in testing our PAC method against the Modulation Index in iEEG data show that we achieve very similar performance in exploratory analyses of PA coupled signals. In Figures 6A, B, the plots on the left half show comodulograms produced using both the Modulation Index (left column) developed by Tort et al., and our method (right column). The right half of the plots show the corresponding multiple comparisons plots for rejection of null hypotheses associated the no coupling for a specific pair of frequencies. For any subfigure, Figures 6A, B, the top row of plots corresponds to the theta-HG coupling and the bottom row corresponds to the theta-HFO coupling. All pixels on the heatmap corresponding to hypotheses that did not pass the multiple comparisons test were set to 0. As we can see in the figure, our method performs comparably to the Modulation Index when used in electrophysiologic recordings in exploratory analyses to try and discover coupling. Note that invasive electrophysiologic recordings has higher SNR compared to noninvasive recordings and thus we use this dataset to see if our method can reliably reproduce coupling in these data. One finding of interest here is also that when performing the multiple comparisons test, our PAC measure always accepted fewer hypotheses in this case no matter the dataset nor multiple comparisons correction procedure. This implies that our method may be better and not accepting hypotheses for which coupled frequencies are only tangentially in the frequency bands being studied. For example, if one were assessing PAC and there existed oscillators at 5–10 and 130–150 Hz, but the frequency bands being studied are 5–10 and 120–140 Hz, our method may be better at not accepting such cases as true coupling.

### 3.6 Joint EEG-EGG recordings

**Figure 7:**
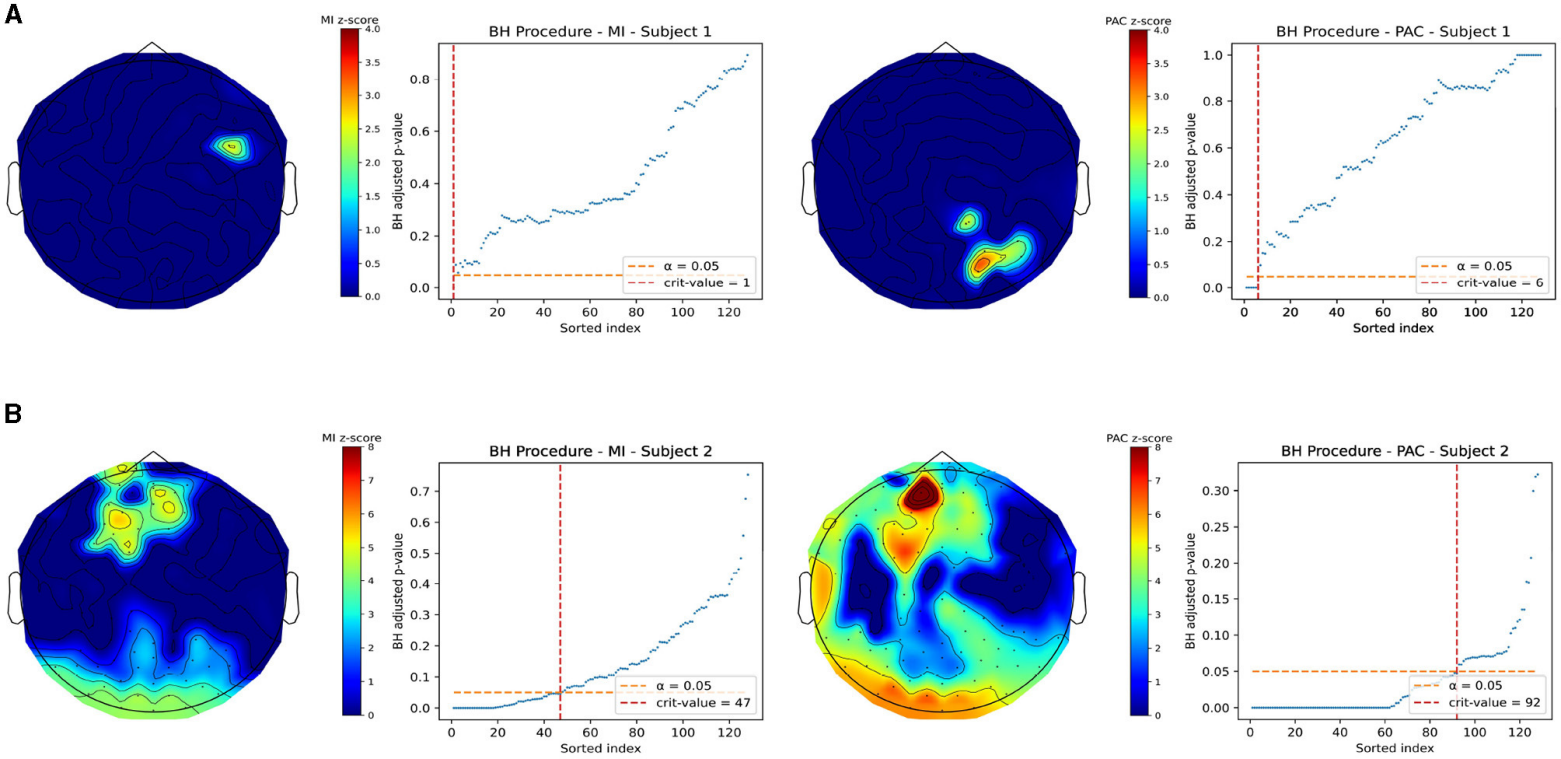
*Z*-scored PAC values for Joint EEG-EGG recordings taken at rest with eyes closed after correction for multiple comparisons. All non-significant channels were set to 0 for image contrast. In both figures, the left column of plots corresponds to the MI and the right column corresponds to our PAC measure. **(A)** Example subject in which our measure and the MI do not match well and show differing areas of significant coupling. **(B)** A different subject in which regions of coupling match well with the MI and our measure. This may indicate a differential ability in our measure and MI in detecting coupling.

Electrophysiological gut-brain PAC was estimated from joint recordings in two subjects at rest with eyes closed. Figure 7 demonstrates topoplots of gut-brain PAC using both MI and our measure in both subjects. In any subfigure, the left half of the plots correspond to the MI and the right half of the plots correspond to our PAC measure. In addition. Each plot is a heatmap of *z*-scored PAC values, where the locations that did not pass multiple comparisons testing were zeroed out for contrast. The multiple comparisons plots are displayed next to each topoplot. Each subfigure, Figures 7A, B, are separate subjects. These topoplots are a top-down view on the brain, where the triangle on the top of the plot represents the nose, thus the front of the brain, and the lobes on the side represent the ears. Note that for visualization we removed electrodes from the 128-channel system that correspond to locations below the ear and nose respectively, which are not significantly near cortical regions of interest.

Figure 7A demonstrates a subject for which the gut-brain PAC at rest, as quantified by our measure and MI, differ significantly in which regions of the brain are highlighted as regions of significant coupling with the gastric slow wave. Note that in each row, the topoplots are beside the corresponding multiple comparisons procedure plot and any The MI demonstrates right parieto-temporal regions of the brain, whereas our measure picked out a small part of the right occipital lobe as a region of active coupling. On the other hand, Figure 7B demonstrates a subject in which there is stronger agreement between the MI and our measure. Both measures pick out occipital regions as well as central frontal regions of the brain as active areas of coupling. It is interesting to point out that while across subjects the modulation index differed in its selection of brain regions coupled to the gut at rest, our measure was able to consistently pick out regions of the occipital lobe as highly coupled to the gut. Note, that these findings are exploratory and should be taken as such, since the limited existing data are shown here. In the discussion we will further explore the potential implications for these differences in finding.

### 3.7 Time-varying PAC in sleep spindle and MMN data

**Figure 8:**
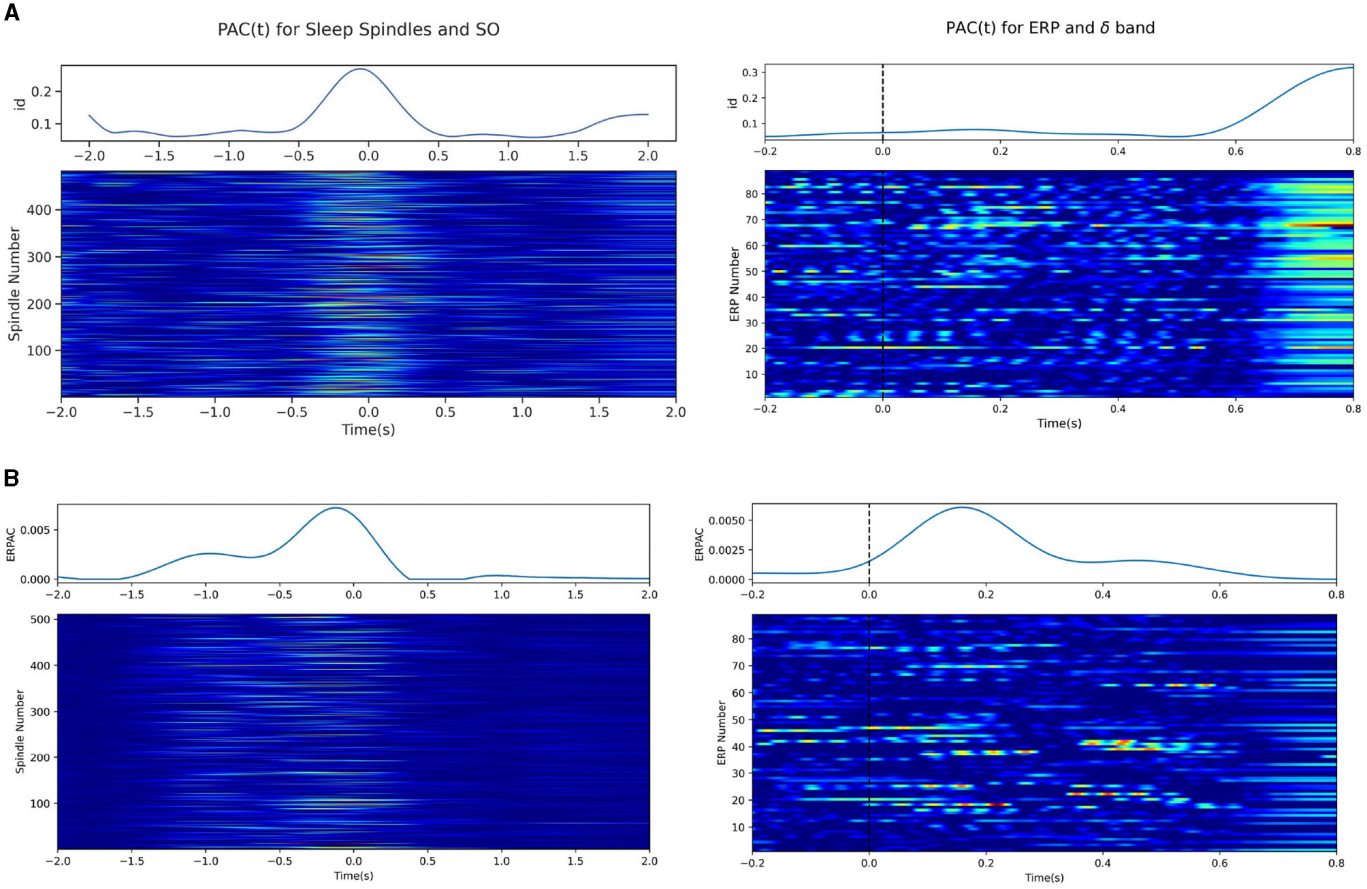
Time-resolved PAC measures applied on sleep spindle data. Each row in the heatmap represents the time-resolved PAC for a single sleep spindle, where each spindle has its peak centered at time 0. The top line plots demonstrate the average time-resolved PAC for each of the ERPAC and idPAC methods. **(A)** (left) idPAC using the gamma GLM was applied to the sleep spindles and demonstrates between sleep spindles (12–16 Hz) and slow oscillations (0.1–1.25 Hz). (right) idPAC applied to the MMN data between the delta band (0.5–4.0 Hz) and the high-frequency component (8.0–15.0 Hz). **(B)** ERPAC calculated on the sleep spindle (left) and MMN data (right).

Experiments assessing the potential for idPAC and ERPAC using our method were done in sleep spindle and MMN data as described in the Sections 2.7.3 and 2.7.4. We note here that instead of a standard lowpass filter, we opt for a Gaussian filter with a standard deviation of 0.08s, to match the cutoff as desired in Section 2.6.3. This is to mitigate edge effects, since the MMN data was pre-epoched into small intervals. In Figure 8A we demonstrate the successful application of idPAC to both the sleep spindle data and the MMN ERPs. Each row of the heatmap in Figure 8A represents one epoch/trial of spindle/ERP data and the color intensity represents the idPAC value. For the sleep spindle data (left) the peak of the spindle was located at time 0, and we note that the idPAC consistently estimated high PAC values between the SO and sleep spindle during the occurence of a sleep spindle. The blue trace above the heatmap is a represenation of the mean idPAC across all trials and demonstrates this same phenomenon. Similarly in the MMN data (right) we notice that the peak in PAC value for each trial occurs ~100–150 ms post-stimulus, which is when we expect the peak of the ERP to occur. The orange dotted line shows the mean ERP across trials and the blue line shows the mean idPAC(t) that highly correlates with the ERP and suggests that the delta band phase may have a significant role in generating ERPs in response to oddball stimuli.

In Figure 8B, we show similar plots for the ERPAC methodology. For the sleep spindle data, we notice a very similar response in the ERPAC as compared to the mean idPAC(t) trace, which is also reflected in the heatmap showing the PAC for each spindle associated with each time point. We note, however, that the ERPAC case may be estimating some spurious PAC pre-spindle as evidenced by the larger PAC values before the spindle peak. On the right, the MMN data also show a peak in ERPAC around the stimulus; however, the ERPAC heatmap tends to show less spurious activation than its idPAC counterpart. Upon close inspection, the idPAC does increase relative to its surroundings when the ERP occurs, but suffers from large edge effects at the end, which wash out the smaller changes in the middle.

To explain the differences between the two techniques, we note that while the heatmaps for idPAC and ERPAC look similar, the philosophies behind the generation of each plot differ. In the idPAC case, the idPAC is calculated for each trial/epoch and is thus a one-shot estimation of the time-varying PAC for each trial/epoch. However, in the ERPAC case, the idPAC is calculated for each column/time point. Here aggregate statistics from multiple similar trials are taken to estimate one grand ERPAC for the phenomenon and the heatmaps are the results from the aggregate statistics around each time point being “projected” back onto each trial/epoch. Note, that while these one-shot estimators may be interesting in the case of data with transient phenomena that may not be easily time-locked/replicable, in the MMN case it does generate large spurious couplings and may be subject to edge effects.

## 4 Discussion

In this paper, we demonstrate that by using a principled method of fitting the joint distribution on amplitude and phase, we can use mutual information as a reliable measure of PAC. This builds upon our previous work to generate more representative distributions on non-negative random phenomena: the gamma distribution as opposed to the exponential distribution. This allows for us to better capture the conditional distribution of amplitude given phase, which has most of its mass away from zero as compared to the exponential distribution (Perley and Coleman, 2022). As shown in Figure 3, our method outperforms the major gold-standard methods of PAC. We believe that the added statistical rigor from explicitly modeling the joint distribution allows us to more flexibly capture the nature and strength of the relationships between phase and amplitude. Our assessment of the GOF also gives us confidence in our model design and validates our choice of a parametric method of mutual information estimation, as this implies we can obtain nearly unbiased estimates. This also demonstrates the need for more advanced measures of CFC. In the future, we envision that our modeling paradigm can be extended appropriately to other forms of CFC such as phase-phase coupling, amplitude-amplitude coupling, and phase-frequency coupling.

In neural data, we have also demonstrated our measure's ability to pick out known PAC and potentially discover new PAC. In the invasive mouse EEG recordings, the high SNR in the makes it easy to pick out PAC in data even using conventional methods. Given these prior findings, we validated our method against the conventional methods and found high correspondence between the produced comodulograms from the data. We also show the potential of our method to be less sensitive to leakage in frequency bands since it accepts fewer hypotheses than the MI after multiple comparisons. One point of discussion that we think is useful is on our use of the FOOOF package to estimate the upper bound of the range of frequnecies to scan over. We do this since we wanted to replicate prior findings, however, a more powerful use of the tool would be to estimate the locations of all the spectral peaks and only compute PAC between pairs of frequencies that have spectral peaks. One could then use some form of surrogate data analysis to confirm the significance of those PAC findings. Since comodulograms can be considered as a multiple hypothesis testing problem, using FOOOF in this way could reduce the number of hypotheses to test. Combined with our more powerful method of quantifying PAC, this may make it easier to more powerfully detect positive results. On the other hand, in joint EEG-EGG recordings (a low SNR setting) we find that there are inconsistencies between the MI and our measure. Since our measure outperforms conventional PAC in simulated data for modest coupling, this suggests that the inconsistencies in our method and MI can potentially be attributed to spurious findings in the MI. Preliminarily, our method also seems to show more consistency across subjects. This may point to the ability of our method to pick out more robust coupling “motifs” across human subjects. However, these claims have yet to be validated in larger datasets and are the subject of future work.

We also demonstrate the potential to use our model fitting procedure to infer a measure of time point-by-time point resolved PAC using information density, and an ERPAC-like paradigm to estimate time-varying PAC for brain responses to the same stimuli. Note that while both of these methods seem to be able to estimate aggregate time-varying PAC for multiple trials of similar phenomena, it may be prudent to opt for the ERPAC method to avoid potential overfitting of the data which may cause spurious PAC like the MMN data in Figure 8. On the other hand, the idPAC phenomenon may be more appropriate for situations where one is looking at long time recordings of coupled electrophysiology where it may not necessarily be appropriate to epoch data into similar chunks/phenomena.

On the practical end, our model fitting procedure is convex and therefore easier to implement rigorous procedures for the average user and therefore we envision the wide distribution of our measure. However, it also loses some of the computational efficiency of measures such as the Modulation Index with simpler estimation procedures. As it stands now, our measure is more suited for precise offline calculations of PAC done post-experiment. Other methods might be more suited for online calculation of PAC for potential use in real-time biofeedback, monitoring, or stimulation. To close this gap, we plan to further develop our method to forgo the use of traditional general convex solvers for the iteratively reweighted least squares (IRLS) method to speed up performance.

It may also be interesting to note that future implementations of our method may extend to include gamma mixture models to capture multimodal conditional distributions for a potentially richer fit on the joint distribution. However, this method will likely end up nonconvex and lose some of the associated advantages. As an aside, we also note that in this work we use the uniform prior on phase, but one can easily extend this to use an empirical prior drawn from the data if the marginal distribution on phase does indeed deviate significantly from a uniform distribution.

We would be remiss not to note some cautions when it comes to the interpretation of idPAC and PAC more generally in a neuroscientific context. One issue to note with the idPAC measure is the one shown in Figure 8A on the right for the MMN data. The idPAC shows large values of coupling across each trial toward the end of the recording, even though it does show a modest increase relative to its surroundings around the timing of the ERP. This serves as an example for potential issues with the technique. One of the issues as noted in Aru et al., is that when trialwise data is limited to only a small section around the trial, filtering may cause edge effects that interfere with the results (Aru et al., 2015). However, another reason may lie in the fact that using one-shot measures, may be more sensitive to non-coupled phenomena. In light of this, we recommend selecting the ERPAC measure of coupling in cases where repeatable trial-wise data are collected. In such cases where the data are not easily repeatable and time-varying transients in coupling are of interest, we encourage users to employ some form of surrogate data analysis to better confirm the effects of coupling in data.

On the note of PAC analysis in general, it has been noted by groups such as Aru et al. (2015) and Gerber et al. (2016), that PAC analysis, when done without proper care, and lead to spurious findings that may not have any neuroscientific basis. For example, Gerber et al. (2016), showed that in simulation that non-sinusoidal periodic activity can give rise to spurious coupling, while in Aru et al. (2015), they show that interpretability of PAC heavily depends on the care taken in preprocessing and inspection of the data. Another point to discuss, is that the FOOOF tool is very useful in determining the existence of spectral peaks and relevant bandwidths of those peaks: criteria listed in Aru et al., for rigorous analysis of PAC. In light of these findings, we urge readers to be cognizant that, while we believe our method performs better in detection of PAC as compared to other methods, it may still be subject to detection of spurious results if care isn't taken to inspect the data closely. Aru et al., presents a non-exhaustive list of checks that researchers should follow before being confident that one's analysis may be on the correct path.

In conclusion, we have demonstrated a promising method for quantification of PAC in electrophysiological recordings. In the future, we imagine that with the dissemination of our method, we may enable the community to conduct more rigorous studies in the discovery of basic and clinical neuro-electrophysiology.

## Data availability statement

The raw data supporting the conclusions of this article will be made available by the authors, without undue reservation.

## Ethics statement

The studies involving humans were approved by Stanford University Institutional Review Board. The studies were conducted in accordance with the local legislation and institutional requirements. The participants provided their written informed consent to participate in this study.

## Author contributions

ASP: Writing—review & editing, Writing—original draft, Visualization, Validation, Software, Methodology, Investigation, Formal analysis, Data curation, Conceptualization. TPC: Writing—review & editing, Supervision, Resources, Project administration, Methodology, Funding acquisition, Conceptualization.
